# Patterns and clinical manifestations of tuberculous myocarditis: a systematic review of cases

**DOI:** 10.11604/pamj.2015.21.118.4282

**Published:** 2015-06-12

**Authors:** Brian Nyasani Michira, Faraj Omar Alkizim, Duncan Mwangangi Matheka

**Affiliations:** 1 School of Medicine, University of Nairobi, Nairobi 00100, Kenya; 2 Department of Medical Physiology, University of Nairobi, Nairobi 00100, Kenya

**Keywords:** Tuberculosis, myocarditis, sudden cardiac death

## Abstract

Tuberculosis is a rare cause of myocarditis. It is however associated with a high mortality when it occurs and is often diagnosed at post-mortem. Tuberculous myocarditis prevalence in males is twice that in females. Most of the reported cases of tuberculous myocarditis are predominantly in immunocompetent patients. Out of the reported fatalities (sudden cardiac deaths), eighty one percent (81%) occur in the ‘young’ patients (below 45years). Antituberculosis drug therapy does not appear to offer mortality benefit against sudden cardiac deaths.

## Introduction

Tuberculosis is endemic in Kenya and is more commonly seen in immunosuppressive states such as HIV/AIDS. Tuberculous myocarditis is however an unusual sequelae, with its prevalence having been reported at 0.14%, 0.2% and 2% in various series [1–3]. Tuberculous myocarditis is mostly diagnosed in association with pericarditis and pericardial effusion. It is mostly asymptomatic but may present with ventricular fibrillation, long QT syndrome, congestive heart failure, dilated cardiomyopathy and even sudden cardiac arrest.

An anatomical predilection for the right-sided mediastinal lymph nodes has been described in this condition, making the right side of the heart the most vulnerable area of the myocardium owing to the potential for contiguous spread [4]. Three distinct forms of myocardial involvement are recognized: nodular tubercles of the myocardium (characterized by central caseation); miliary tubercles of the myocardium (resulting from hematogenous spread); and an uncommon diffuse infiltrative type associated with tuberculous pericarditis (characterized microscopically by giant cells and lymphocytes) [5, 6]. The myocardium may be involved by hematogenous spread, by retrograde lymphatic spread from mediastinal lymph nodes or by direct invasion from the pericardium [7, 8]. The diagnosis can be made by myocardial biopsy if clinical suspicion is strong and echocardiographic findings are suggestive.

Published reports of tuberculosis related sudden deaths between the years 1966 and 2000 showed that most of the cases were due to tuberculous bronchopneumonia (64%) and massive haemoptysis (30%), with a minority of cases due to tuberculous myocarditis [9]. Having a low incidence rate coupled with an insidious onset and progression, TB myocarditis is commonly undiagnosed ante mortem. Studies have shown that many such cases are thereby allowed to progress leading to death and late diagnosis at postmortem.

The low incidence, late diagnosis and under-reporting have with time created a knowledge gap among health care workers. This review therefore seeks to restore awareness among the practitioners, to promote a high index of suspicion for early diagnosis, and thereby timely management of tuberculous myocarditis.

## Methods

A PubMed search using the keywords “Tuberculous myocarditis” and “Tuberculosis myocarditis” yielded one hundred and thirty six (136) articles; of which 23 were included as highly relevant to this review with no geographical focus. Articles published between the years 2000 and 2013 were included in the review, which was consistent with an era of revamped efforts and global policies to control and prevent tuberculosis in societies. Articles included were limited to cases of tuberculosis complicated with myocarditis or perimyocarditis. Attention was also given to whether the patients were free from preexisting cardiac comorbidities and to rule out primary tumors or metastases, idiopathic giant cell myocarditis and other granulomatous lesions. Articles without sufficient information to address these issues were excluded from this review. The variable clinical presentations, progression and prevalence of tuberculous myocarditis were reviewed.

### Current status of knowledge

The tuberculous myocarditis cases reviewed are predominantly in immunocompetent patients (Table 1). Concomitant pulmonary infection was reported in 9 (56%) of the cases. Concomitant pericarditis was recorded in 7 (43%) of the cases. Involvement of other extrapulmonary sites apart from the heart was recorded in 9 (56%) of the cases. Isolated cases of tuberculous myocarditis without involvement of any other organs were 4 (25%) of all reviewed cases. Eleven (68%) of the patients responded well to antituberculosis treatment with 5 (31%) fatalities from sudden cardiac death recorded (Table 1).

**Table 1:** Pattern and distribution of tuberculous myocarditis

Author et al	Age (yrs)	Sex	Immunological status	Pulmonary involvement	Extrapulmonary site involved	Area of heart affected	Outcome of therapy
Maeder [4]	22	M	Competent	Yes	Mediastinal lymph nodes	R. Atrium	Responsive
Gautam [6]	33	M	Competent	Yes	Lymphadenitis	Biventricular	Responsive
Agarwal [7]	25	F	Competent	Yes	None	Global chamber enlargement	Fatal (SCD)
Agarwal [8]	28	M	Competent	Yes	None	Global chamber enlargement	Responsive
Jokhdar [10]	28	F	Competent	Yes	None	R. Atrium L. Ventricle	Responsive
Marano [11]	65	M	Competent	No	Lymphadenitis	L. Atrium R. Ventricle	Responsive
Gulati [12]	12	M	Competent	No	None	Biventricular	Responsive
Trilla [13]	26	M	Competent	Yes	Skin abscesses	R. Atrium	Responsive
Roubille [14]	53	M	Competent	Yes	Systemic spread	L. Ventricle	Responsive
Khurana [15]	30	M	Competent	No	None	R. Atrium R. Ventricle	Responsive
Dada [16]	25	M	Competent	No	None	L. Ventricle	Fatal (SCD)
Silingardi [17]	33	F	Competent	Yes	Spleen, liver, lymph nodes	L. Ventricle	Fatal (SCD)
Amonkar [18]	65	F	Competent	No	Liver	Biventricular	Fatal (SCD)
Biedrzycki [19]	20	F	Competent	No	None	L. Ventricle	Fatal (SCD)
Desai [20]	28	M	Competent	No	Ileocecal, mesenteric and mediastinal lymph nodes	L. Ventricle	Responsive
Diaz [21]	32	M	Compromised	Yes	Liver	Unreported	Responsive

Legend: SCD= Sudden Cardiac Death, M= Male, F= Female, R= Right, L= Left

Tuberculous myocarditis was predominantly reported in the ‘young’ (below 45yrs), accounting for 81% of the reports. Twice as many males were affected with tuberculous myocarditis as females. There was also a predilection to site of the heart involved, with the left ventricle commonly affected (68%). The right ventricle was affected in 43% of the cases; the right atrium in 37% of the cases; the left atrium in 18% of the cases (Table 1).

Of the reported fatalities (sudden cardiac deaths), 80% of the fatalities were females with left ventricular involvement seen in all of these cases. All of the four isolated cases of myocarditis with no other organ involvement were in ‘young’ (below 45yrs) patients, mainly among males (75%). The most commonly involved extrapulmonary site was the mediastinal lymph nodes followed by the liver (Table 1).

Electrical conduction abnormalities in the myocardium seem not to be entirely dependent on serum electrolyte levels. In the case report by Agarwal et al [8], S3 heart sound with sinus tachycardia were recorded with a high serum Ca++ of 9.9 mEq/L, whereas in the case report by Jokhdar et al [9], S3 apical gallop with sinus tachycardia were recorded with a low serum Ca++ of 6.3 mg/dL. Tuberculous myocarditis seems to only affect the mitral and tricuspid valves causing valvular incompetence. Valvular stenosis is however not observed. The semilunar valves are unaffected (Table 2). The antituberculosis regimen was effective for tuberculous myocarditis, improving clinical picture and reducing hospital stay but had no mortality benefit.

**Table 2:** Clinical manifestations of tuberculous myocarditis

Author et al	Clinical Manifestations
Maeder [4]	Large mediastinal mass infiltrating the right atrium and adjacent vasculature, cardiomegaly, arrhythmias, right bundle branch block, sinus tachycardia.
Gautam [6]	Refractory ventricular tachycardia.
Agarwal [8]	Congestive cardiac failure, cardiomegaly.
Jokhdar [10]	Congestive cardiac failure.
Marano [11]	Left atrium and right ventricle infiltration by lesions, arrhythmias, *diagnosis reached ex juvantibus.
Gulati [12]	Infiltrative nodular masses in outer myocardium and pericardium involving both ventricles and right atrium.
Trilla [13]	Mass adherent to the right atrium.
Roubille [14]	Mimicking an acute coronary syndrome with elevated troponin Ic and negative T waves on ECG.
Khurana [15]	Cardiomegaly.
Dada [16]	Sudden cardiac death.
Silingardi [17]	Sudden cardiac death.
Biedrzycki [19]	Sudden cardiac death.
Desai [20]	Congestive cardiac failure.
Diaz-Peromingo [21]	Long QT syndrome.
Afzal [22]	Pericarditis, cardiac tamponade.
Rodriguez [23]	Calcified submitral mass in the free wall of left ventricle, normal sinus rhythm.
Jagia [24]	Myocardial tuberculoma of the right atrium with accompanying intracerebral tuberculoma.
Agarwal [25]	Cardiomegaly.
Akhulaifi [26]	Mass encroaching the right atrium (myxoma and malignancy were ruled out on biopsy).
Brar [27]	Congestive cardiac failure.
Mteirek [28]	(a) Case 1: Myopericarditis (b) Case 2: Pseudo-infarction complicated with cardiogenic shock.

## Conclusion

Myocarditis related to a recent tuberculosis infection has scarcely been reported in practice. This could be due to the insidious nature of majority of cases and thereby many cases are diagnosed at postmortem. Almost all cases are responsive to anti-tuberculosis drug therapy, but the risk of sudden cardiac death from this condition is not reduced by the treatment hence the need for close monitoring and symptomatic relief of cardiac abnormalities when reported. In tuberculosis endemic areas, a high index of suspicion is necessary in patients presenting with unexplained non-ischemic arrhythmias, congestive heart failure or cardiogenic shock.
